# QuickBundles, a Method for Tractography Simplification

**DOI:** 10.3389/fnins.2012.00175

**Published:** 2012-12-11

**Authors:** Eleftherios Garyfallidis, Matthew Brett, Marta Morgado Correia, Guy B. Williams, Ian Nimmo-Smith

**Affiliations:** ^1^Wolfson College, University of CambridgeCambridge, UK; ^2^Medical Research Council Cognition and Brain Sciences UnitCambridge, UK; ^3^Henry H. Wheeler Jr. Brain Imaging Center, University of CaliforniaBerkeley, CA, USA; ^4^Wolfson Brain Imaging Centre, University of CambridgeCambridge, UK

**Keywords:** tractography, diffusion MRI, fiber clustering, white matter segmentation, dimensionality reduction, clustering algorithms, DTI

## Abstract

Diffusion MR data sets produce large numbers of streamlines which are hard to visualize, interact with, and interpret in a clinically acceptable time scale, despite numerous proposed approaches. As a solution we present a simple, compact, tailor-made clustering algorithm, QuickBundles (QB), that overcomes the complexity of these large data sets and provides informative clusters in seconds. Each QB cluster can be represented by a single centroid streamline; collectively these centroid streamlines can be taken as an effective representation of the tractography. We provide a number of tests to show how the QB reduction has good consistency and robustness. We show how the QB reduction can help in the search for similarities across several subjects.

## 1 Introduction

Following the acquisition of diffusion MR scans, processes of reconstruction and integration are performed to create a *tractography* – that is to say a dataset composed of streamlines, which are sequences of points in 3D space. Irrespective of the types of reconstruction and integration, a tractography can contain a very large number of streamlines (up to 10^6^) depending principally on the number of seed points used to generate the tractography but also on how the tractography propagation algorithm handles voxels with underlying fiber crossings.

The size of these tractographies makes them difficult to interpret and visualize. A clustering of some kind seems to be an obvious route to simplify the complexity of these data sets and provide a useful segmentation. As a result, during the last 10 years there have been numerous efforts by many researchers to address both unsupervised and supervised learning problems of brain tractography. As far as we know all these methods suffer from low time efficiency, i.e., they are very slow when used in practice.

In the tractography literature we can find approaches which use unsupervised and/or supervised learning algorithms to create bundles, i.e., groupings of streamlines with similar spatial and shape characteristics. In supervised learning the data sets are divided into a training and a test set. For the training set, experts will have provided anatomical labels for a set of manually segmented streamline bundles. Those bundles will now correspond to tracts, e.g., the corticospinal tract or the arcuate fasciculus. The task then will be to identify similar structures amongst the unlabeled streamlines in the test set.

In unsupervised learning the focus is on creating a partitioning of the streamlines without knowing any labels. In this work we used unsupervised learning to reduce in a simple and efficient way the number of streamlines and make manual segmentation of bundles and tractography exploration less time consuming tasks. By the term *bundle* we mean here streamlines which are in close proximity according to a streamline-based distance, therefore, they have similar spatial and shape characteristics and not necessarily direct correspondence to neuroanatomical bundles (tracts).

We believe that a complete unsupervised method cannot directly create anatomically valid bundles without extensive prior information preferably from experts or atlases. This is because anatomical bundles differ considerably both in lengths and in shape (see Schmahmann and Pandya, 2009) and there is not a single threshold which can cluster a full dataset with anatomical correspondence (see Guevara et al., 2010). Furthermore, if one uses such extensive priors then it will be more suitable to use supervised learning algorithms. Therefore, we propose that unsupervised learning should be used primarily for dimensionality reduction or simplification of these large data sets. This is the focus of the approach that we propose here.

Most clustering (unsupervised learning algorithms) are in the best case of complexity $O\text{(}N^{2}\text{)}$ where *N* the total number of streamlines: they require the calculation of all pairwise distances between streamlines in order to create a distance matrix. We found that most authors tried to create these distance matrices as input to hierarchical clustering (see Moberts et al., 2005; Zhang and Laidlaw, 2005; Tsai et al., 2007; Zhang et al., 2008; Jianu et al., 2009; Guevara et al., 2010, 2012; Visser et al., 2011) or spectral clustering (see Jonasson et al., 2005; O’Donnell and Westin, 2007; O’Donnell et al., 2009; Ziyan et al., 2009) or affinity propagation (see Leemans and Jones, 2009; Malcolm et al., 2009). However, they had to restrict themselves to only a subset of the complete dataset because the calculations were heavy and the distance matrix too big for computers with standard memory capacity. For example, O’Donnell and Westin (2007) used only 10,000 streamlines (∼10% of the initial tractography) and Visser et al. (2011) used recombinations of 10,000 streamlines. We give here an example of how large that distance matrix can be if used with a full dataset. For just 100,000 streamlines 38 GBytes are required, and for 1 million streamlines 3.6 TBytes of memory are required (4 bytes floating point precision).

Other clustering methods have also been proposed that use graph theoretic approaches (see Brun et al., 2004; Gerig et al., 2004; El Kouby et al., 2005), *k*-nearest neighbors (see Ding et al., 2003; Moberts et al., 2005), Gaussian processes (see Wassermann et al., 2010), hierarchical dirichlet processes (see Wang et al., 2011), currents (see Durrleman et al., 2009, 2011), adaptive mean shift (see Zvitia et al., 2008, 2010), *k*-means (see El Kouby et al., 2005), and expectation maximization (see Maddah et al., 2008). Although these methods often try to avoid distance matrices they still suffer from low time efficiency. All the same, if clustering is to be used in clinical practice or to make neuroscientists’ analysis more efficient and practical we need algorithms that can provide useful clusters and cluster descriptors in acceptable time. None of the papers covered in this literature review provide a solution for this issue of efficiency and most of the methods would require from many hours to many days to run on a standard sized dataset.

In fact tractographies have high levels of redundancy with many similar streamlines. Our approach is to take advantage of this to reduce the size and dimensionality of the data sets as a precursor for higher complexity classification and/or input from experts. To address these key issues of time and space we present a stable, generally linear time clustering algorithm that can generate meaningful clusters of streamlines in seconds with minimum memory consumption. Our approach is straightforward and we do not need to calculate all pairwise distances unlike most existing methods. Furthermore we can update our clustering online, i.e., as and when new data points become available. In this way we can overcome the previous barriers of space and time.

We show that QuickBundles can generate these clusters many times much faster than any other available method, and that it can be used to cluster from a few hundred to many millions of streamlines.

**Figure 1:**
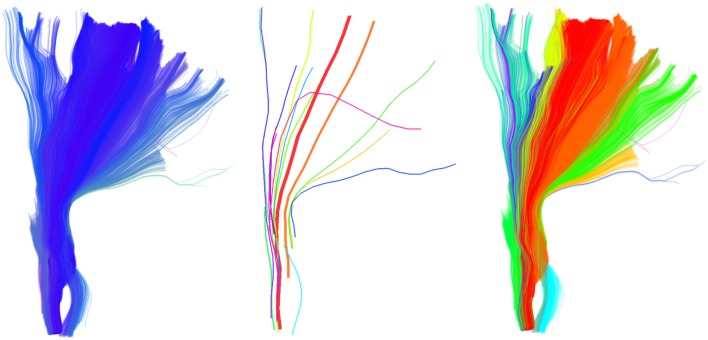
**Part of the CST bundle (left, in blue) consisting of 11,041 streamlines**. At first glance it looks as though all streamlines have a similar shape, and possibly converge toward the bottom, and fan out toward the top. However, this is a misreading caused by the unavoidable opaque density when all the streamlines are visualized. QB can help us see the finer structure of the bundle and identify its elements. In the middle we see the 16 QB centroid streamlines of the CST. We can now clearly observe that several parts which looked homogeneous are actually broken bundles or bundles with rather different shapes. On the right panel we see the streamlines colored according to their cluster label. The clustering threshold used here was 10 mm.

We think that there is no current unsupervised anatomical segmentation method that can have general usability without expert knowledge integration. Nonetheless, neuroanatomists often disagree on definition of major structures or on which streamlines correspond to actual tracts (Catani et al., 2005; Makris et al., 2005; Mori et al., 2005; Frey et al., 2008; Schmahmann and Pandya, 2009; Verstynen et al., 2011; Fernandez-Miranda et al., 2012). What we propose is to use QuickBundles to simplify the tractography at the level where such distinctions are not an issue (see Figure 1). On top of these simplifications we can use other methods of higher complexity much more efficiently.

## 2 Materials and Methods

### 2.1 Streamline distances and preprocessing

A wide range of approaches have been taken in the literature for representing or coding for tractographies (Chung et al., 2010; Guevara et al., 2010). The approach we have taken with streamline coding has gone in parallel with the selection of appropriate functions for inter-streamline distances. Numerous inter-streamline distance functions have been proposed (Ding et al., 2003; Zhang and Laidlaw, 2005; Maddah et al., 2007). The most common is the Hausdorff distance (Corouge et al., 2004; and many other studies). There are two primary disadvantages of this function: it ignores the sequential nature of the streamlines and treats each streamline simply as a cloud of points, and its computation requires every point on the first streamline to be compared with every point on the second streamline, and vice versa. Thus the Hausdorff distance requires the calculation O(KL) inter-point distances when comparing streamlines of *K* and *L* points.

**Figure 2:**
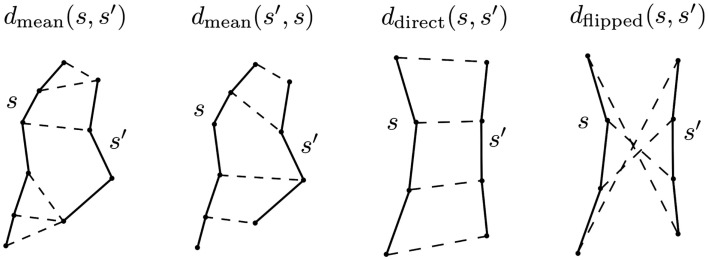
**The principal distance used in this work is minimum average direct-flip distance MDF = min(*d*_direct_, *d*_flipped_) which is a symmetric distance function that can deal with the streamline bi-directionality problem; it works on streamlines which have the same number of points**. Another distance we use is the mean average distance which is again symmetric but does not require the streamlines to have the same number of points: MAM_mean_ = (*d*_mean_(*s*, *s*′) + *d*_mean_(*s*′, *s*)/2 [see equation (5)]. In this figure the components of both distances are shown; the streamlines are drawn with solid lines, and then with dashed lines we connect the pairs of points of the two streamlines whose distances contribute to the overall metric. Note that we cannot calculate the MDF between the streamlines on the left of the figure because they have different numbers of points.

For these reasons we have opted to use a rather simpler symmetric distance function (Garyfallidis et al., 2010; Visser et al., 2011) which we call the minimum average direct-flip (MDF) distance, see equation (1). MDF can be applied only when both streamlines have the same number of points, see Figure 2. Therefore we assume from now on that an initial discretization of streamlines has been applied, so that all streamlines have the same number of points *K*, and all segments of each streamline have equal length. This is achieved by a simple linear interpolation.

As it has no preferred orientation, a streamline *s* = [*s*_1_, *s*_2_, …, *s_K_*] is equivalent to two ordered polylines: *s* = [*s*_1_, *s*_2_, …, *s_K_*] in ℝ^3^ and its flipped version *s^F^* = (*s_K_*, *s*_*K*−1_, …, *s*_1_). With this notation the direct, flipped and MDF distances are defined as follows:

$$\begin{array}{llll} d_{\text{direct}}\left(s,t\right)=d\left(s,t\right) & =\frac{1}{K}\overset{K}{\underset{i=1}{\sum }}\;|s_{i}-t_{i}|, & & \\ d_{\text{flipped}}\left(s,t\right) & =d\left(s,t^{F}\right)=d\left(s^{F},t\right), & & \\ \text{MDF}\left(s,t\right) & =\min\left(d_{\text{direct}}\left(s,t\right),d_{\text{flipped}}\left(s,t\right)\right). & & \text{(1)} \end{array}$$

Here |*x* − *y*| denotes the Euclidean distance between two points *x* and *y*. The direct distance *d*_direct_(*s*, *t*) between two streamlines *s*, and *t* is the mean of the Euclidean distances between corresponding points. Clearly the MDF distance between two streamlines of *K* points requires the calculation of just 2*K* inter-point distances.

The MDF distance is in fact a metric on the space of streamlines. Obviously MDF distances are non-negative, are zero if and only if the two streamlines are identical, and symmetrical. The triangle inequality is established as follows. Let *s*, *t*, and *u* be three streamlines and assume, without loss of generality, that *d*(*s*, *t*) and *d*(*t*, *u*) actually minimize the corresponding MDF distances MDF(*s*, *t*) and MDF(*t*, *u*). (If not, we replace, e.g., *t* by *t^F^*) Then MDF(*s*, *t*) + MDF(*t*, *u*) = *d*(*s*, *t*) + *d*(*t*, *u*) ≥ *d*(*s*, *u*) (by the triangle inequality for the Euclidean distance) ≥ min(*d*(*s*, *u*), *d*(*s, u^F^*)) = MDF(*s*, *u*).

**Figure 3:**
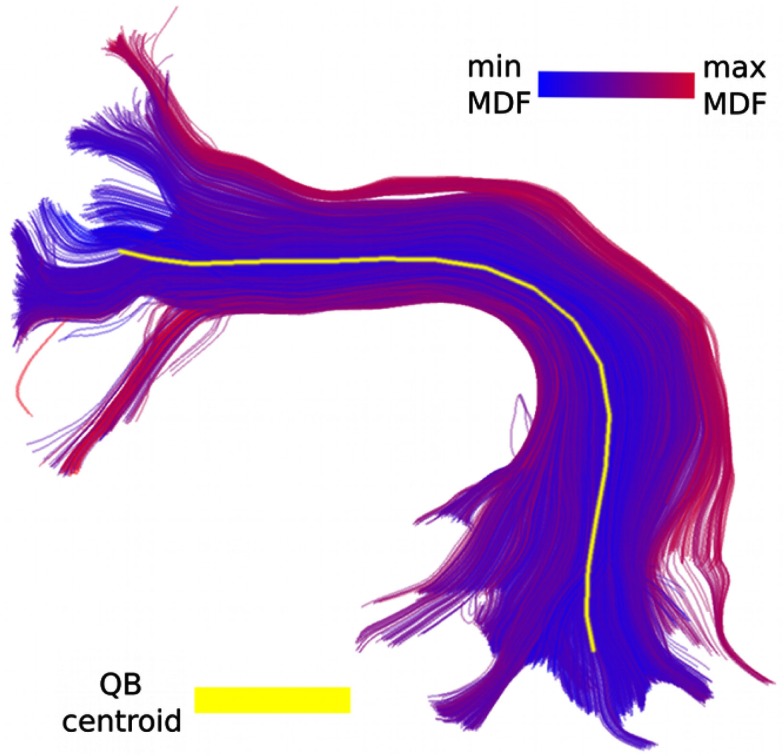
**Color coding shows MDF distances from QB centroid to every other track in the bundle**.

The main advantages of the MDF distance are that it is fast to compute, it takes account of streamline direction issues through consideration of both direct and flipped streamlines, and that its behavior is easy to understand (see Figure 3), from the simplest case of parallel equi-length streamlines to the most complicated with very divergent streamline.

Another advantage of the MDF distance function is that it separates short streamlines from long streamlines; a streamline *s* that is a portion of a streamline *s*′ will be relatively poorly matched on MDF to *s*′. This is consistent with our proposed approach to leave decisions about the status of short versus long streamlines till after applying a clustering. At this later stage one can determine whether there are several similar short streamlines – perhaps reflecting a damaged fiber tract – or localized noise if there are only a few similar streamlines.

A further important advantage of having streamlines with the same number of points is that we can easily do pairwise calculations on them; for example add two or more streamlines together to create a new average streamline. We will see in the next section how streamline addition is a key property that we exploit in the QB clustering algorithm.

Care needs to be given to choosing the number of points required in a streamline (streamline discretization). We always keep the endpoints intact and then discretize into segments of equal lengths. One consequence of short streamlines having the same number of points as long streamlines is that more of the curvature information from the long streamlines is lost relative to the short streamlines, i.e., the short streamlines will have higher resolution. We found empirically that this is not an important issue and that for clustering purposes even discretizing to only *K* = 3 points per streamline can produce useful clusterings (Garyfallidis et al., 2010). Depending on the application, more or fewer points can be used. In the results presented we often use *K* = 12 which achieves a good trade-off between streamline resolution and memory size reduction.

In some later stages in the analysis of tractographies, e.g., for merging clusters, we find a use for Hausdorff-type distance functions which for simplicity we denote as MAM distances – short for Minimum (or Maximum, or Mean) Average Minimum distance (MAM). (In this nomenclature the classical Hausdorff distance is the Maximum Average Minimum distance.) We mostly use the Mean version of this family, see equation (5) but the others are potentially useful as they can weight different properties of the streamlines. As noted above, these distances are slower to compute but they can work with different number of segments on streamlines; this is useful for some applications. The equations below show the formulation of these distances:

$$\begin{array}{llll} d_{\text{mean}}\left(s,s^{\prime }\right) & =\frac{1}{K_{A}}\overset{K}{\underset{i=1}{\sum }}\;d\left(x_{i},s^{\prime }\right), & & \\ d_{\text{min}}\left(s,s^{\prime }\right) & =\underset{i=1,...,K}{\min}d\left(x_{i},s^{\prime }\right),\;\;\text{and} & & \text{(2)}\\ d_{\text{max}}\left(s,s^{\prime }\right) & =\underset{i=1,...,K}{\max}d\left(x_{i},s^{\prime }\right),\;\;\text{where} & & \text{(3)}\\ d\left(x,s^{\prime }\right) & =\underset{j=1,...,K^{\prime }}{\min}|x-x_{j}^{\prime }|. & & \\ {\text{MAM}}_{\text{min}} & =\min\left(d_{\text{mean}}\left(s,s^{\prime }\right),d_{\text{mean}}\left(s^{\prime },s\right)\right) & & \text{(4)}\\ {\text{MAM}}_{\text{max}} & =\max\left(d_{\text{mean}}\left(s,s^{\prime }\right),d_{\text{mean}}\left(s^{\prime },s\right)\right) & & \\ {\text{MAM}}_{\text{mean}} & =\left(d_{\text{mean}}\left(s,s^{\prime }\right)+d_{\text{mean}}\left(s^{\prime },s\right)\right)∕2 & & \text{(5)} \end{array}$$

where the number of points *K* and *K*′ on the two streamlines are not necessarily the same. For the same distance value MAM_min_, MAM_max_, and MAM_mean_ will give different results. For example, MAM_min_ will bring together more short streamlines with long streamlines than MAM_max_, and MAM_mean_ will have an in-between effect. Other distances than *d*(*x*_i_, *s*′) can be used in equations (2 and 3). However, we have not investigated them in this work in relation to clustering algorithms.

### 2.2 The QB algorithm

QuickBundles (QB) is a surprisingly simple and very fast algorithm which can reduce tractography representation to an accessible structure in a time that is linear in the number of streamlines *N*. QB is an extended update on our preliminary work (Garyfallidis et al., 2010).

In QB each item, a streamline, is a fixed-length ordered sequence of points in ℝ^3^, and QB uses comparison functions and amalgamations which take account of and preserve this structure. Moreover each item is either added to an existing cluster on the basis of the distances between the cluster descriptor of the item and the descriptors of the current list of clusters. Clusters are held in a list which is extended according to need. Unlike amalgamation clustering algorithms such as *k*-means (Steinhaus, 1956; MacQueen, 1967) or BIRCH (Zhang et al., 1997), there is no reassignment or updating phase in QB – once an item is assigned to a cluster it stays there, and clusters are not amalgamated. QB derives its speed and efficiency from this idea.

A clustering algorithm needs a measure of distance between two streamlines, and QB uses a particular distance measure that we call minimum average direct flip (MDF). The MDF measure requires that each streamline be resampled to have *K* points. We have described the MDF measure and the resampling in Section 2.1.

QuickBundles stores information about clusters in *cluster nodes*. We index the streamlines with *i* = 1, …, *N* where *s_i_* is the *K* × 3 matrix representing streamline *i*. A cluster node is defined as a triple *c* = (*I*, *h*, *n*) where *I* is the list of the integer indices *i* = 1, …, *N* of the streamlines in that cluster, *n* is the number of streamlines in the cluster, and *h* is the *streamline sum*. *h* is a *K* × 3 matrix which can be updated online when a streamline is added to a cluster and is equal to:

(6) $$h=\overset{n}{\underset{i=1}{\sum }}\;s_{i}$$

where *s_i_* is the *K* × 3 matrix representing streamline *i*, Σ here represents matrix addition, and *n* is the number of streamlines in the cluster. One summary of the cluster node is the centroid streamline *v* where:

(7) v=h∕n

An example of the QB centroid is presented in Figure 3.

Algorithm 1: QuickBundles



**Input:** *T* (=) {*s*_1_, (…), *s_i_*, (…), *s_N_*}, (þeta)
**Output:** *C* (=) [*c*_1_, (…), *c_k_*, (…), *c_M_*]
 # create first cluster
 *c*_1_ (←) ([1],*s*_1,1_)
 *C* (←) [*c*_1_]
 *M* (←) 1
 **for** *i* (=) 2 to *N* **do**
  *t* (←) *s_i_*
  alld (←) **infinity**(*M*) # distance buffer
  flip (←) **zeros**(*M*) # flipping check buffer
  **for** *k* (=) 1 to *M* **do**
   *v* (←) *c_k_*(·)*h*/*c_k_*(·)*n*
   *d* (←) d_direct_(*t*,*v*)
   *f* (←) *d*_flipped_(*t*,*v*)
   # evaluate MDF
   **if** *f* < *d* **then**
    *d* (←) *f*
    flip_k_ (←) 1
   **end if**
   alld_k_ (←) d
  **end for**
  *m* (←) min(alld)
  *l* (←) argmin(alld)
  **if** *m* (<) (þeta) **then**
   # append to current cluster
   **if** flip*_l_* is 1 **then**
     *c*_l_(·)h (←) *c_l_*(·)*h* + *t^F^*
   **else**
     *c_l_*(·)*h* (←) *c_l_*(·)*h* + *t*
   **end if**
   *c_l_*(·)*n* (←) *c_l_*(·)*n* + 1
   **append**(*c_l_*(·)*I*,*i*)
  **else**
   # create new cluster
   *c*_M(+)1_ (←) ([*i*],*t*,1)
   **append**(*C*,*c*_M(+)1_)
   *M* (←) *M*(+)1
  **end if**
 **end for**



The algorithm proceeds as follows. At any one step in the algorithm we have *M* clusters. Select the first streamline *s*_1_ and place it in the first cluster *c*_1_ ← ({1}, *s*_1_, 1); *M* = 1 at this point. For each remaining streamline in turn *i* = 2, …, *N*: (i) calculate the MDF distance between streamline *s_i_* and the centroid streamlines *v_e_* of all the current clusters *c_e_*, *e* = 1, …, *M*, where *v* is defined on the fly as *v* = *h*/*n*; (iii) if any of the MDF values *m_e_* are smaller than a clustering threshold þ, add streamline *i* to the cluster *e* with the minimum value for *m_e_*; *c_e_* = (*I*, *h*, *n*), and update *c_e_* ← (**append**(*I*, *i*), *h* + *s*, *n* + 1); otherwise create a new cluster *c*_*M*+1_ ← ([*i*], *s_i_*, 1), *M* ← *M* + 1.

Choice of orientation can become an issue when adding streamlines together, because streamlines can equivalently have their points ordered 1, …, *K* or be flipped with order *K*, …, 1. A step in QB takes account of the possibility of needing to perform such a flip of a streamline before adding it to a centroid streamline according to which direction produced the MDF value.

The complete QB algorithm is described in formal detail in Algorithm 1. One of the reasons why QB has on average linear time complexity derives from the structure of the cluster node: we only save the sum of current streamlines *h* in the cluster and the sum is cumulative; moreover there is no recalculation of clusters, the streamlines are passed through only once and a streamline is assigned to one cluster only.

### 2.3 The QB representation

One of the major benefits of applying QB to tractographies is that it can provide meaningful simplifications and find features that were previously invisible or difficult to locate because of the high density of the tractography. For example we used QB to cluster part of the corticospinal tract (CST). This bundle was labeled in the data sets provided by PBC (2.5) and it was selected by an expert. The QB representation is clearly shown in Figure 1 where every cluster is represented by a single centroid streamline. To generate this clustering we used a tight threshold of 10 mm. We observe that only a few centroid streamlines travel the full distance from bottom to top and that they are many streamlines that are broken (i.e., shorter than what was initially expected) or highly divergent.

**Figure 4:**
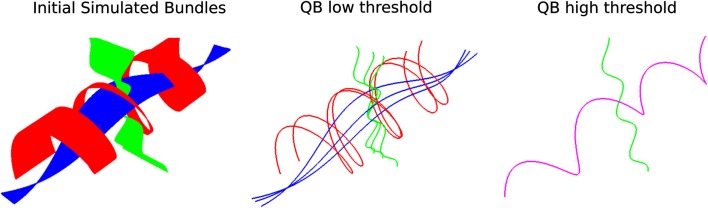
**Left: 3 bundles of simulated trajectories; red, blue, and green consisting of 150 streamlines each**. All 450 streamlines are clustered together using QB. Middle and Right: centroid streamlines using thresholds 1 and 8, respectively. At low threshold the underlying structure is reflected in a more detailed representation. At higher threshold, closer bundles merge together. Here the red and blue bundle have merged together in one cluster represented by the purple centroid streamline.

Another interesting feature of QB is that it can be used to merge or split different structures by changing the clustering threshold. This is shown in Figure 4; on the left we see simulated paths made from simple sinusoidal and helicoidal functions packed together. The color coding is used to distinguish the three different structures. With a lower threshold the three different structures remain separated but when we use a higher threshold the red and blue bundles are represented by only one cluster indicated by the purple centroid streamline.

**Figure 5:**
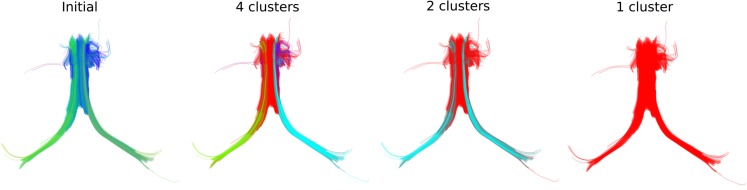
**QB clustering of the Fornix bundle**. The original Fornix (1076 streamlines) is shown on the left panel using a standard orientation colormap. We observe that the Fornix consists of two long distant legs (left and right Fimbria) and a thicker upper part (Body of Fornix). We show here how QB will be able to distinguish the parts of the Fornix at different resolutions. With a 15 mm threshold QB generates 4 clusters shown on the second panel with distinct colors. Here the left and right Fimbria are clearly distinguished from the Body. A last cluster (with blue) exposes a shorter part of the Body which is probably due to noise in the data. With a 18 mm threshold only two clusters are created. Both Fimbria are brought together as one cluster. A property useful for studies which want to use both Fimbria as one. With a 20 mm threshold the entire Fornix is one cluster. This is useful for interactive applications because now the entire Fornix can be described by only one centroid streamline. The streamlines of the Fornix were discretized to have 18 points.

We can see similar effects with real streamlines, for instance those of the fornix shown at the left panel of Figure 5 where we can obtain different numbers of clusters at different thresholds. In that way we can stress thinner or thicker sub-bundles contained inside other bigger bundles.

In order to quantify the dimensionality reduction it achieves we applied QB clustering to the 10 human subject data sets (2.5). The mean data compression (ratio of tractography size to number of QB clusters) was 34.4:1 with a 10 mm threshold and 230.4:1 with a 20 mm threshold.

### 2.4 Comparison of clusterings

We have found rather few systematic ways available in the literature to directly compare different clustering results for tractographies, beyond that of Moberts et al. (2005) who quantified the agreement between a clustering and a “gold standard” tractography labeled by their team. We have used a more symmetrical measure of agreement between two clusterings that do not require a prior labeled dataset. It is called the *optimized matched agreement* (OMA). As with the *adjusted Rand index* (ARI; Moberts et al., 2005), OMA requires the calculation of the *M* × *N* streamline cross-classification matrix *X* = (*x_ij_*). The entries of *X* are the counts of the number of streamlines in all pairwise intersections of clusters, one from each of the two clusterings. If 𝒜 = {*A_i_* : *i* = 1, …, *M*} and ℬ = {*B_j_* : *j* = 1, …, *N* are the two clusterings, then *x_ij_* = |*A_i_* ∩ *B_j_*|. As there is no *a priori* correspondence or *matching* between the clusters in 𝒜 and those in ℬ, and vice versa, we need to find one empirically. If *j* = *π*(*i*) is a possible matching then the corresponding *matched agreement* is $\text{MA}\left(\pi \right)=\sum_{i=1}^{M}\;x_{i,\pi \left(i\right)}$. A matching *π* that yields OMA by maximizing MA(*π*) can be found using the Hungarian Algorithm (Kuhn, 1955). The interpretation of the OMA statistic is analogous to that of the well-known Kappa measure of inter-rater agreement (Altman, 1995), with the range 61–80% corresponding to a good strength of agreement.

We will use OMA to compare the different clusterings that arise when the streamlines in the tractography are shuffled. However this statistic has its limitations. Not only are there considerable computational overheads in calculating the cross-classification matrix, there is also a fundamental disadvantage because they do not work with clusterings of different tractographies. Being able to compare results of clusterings across brains is crucial for creating stable brain imaging procedures, and therefore it is necessary to develop a way to compare different tractography clusterings on different sets of streamlines from the same or different subjects.

Although we recognize that these are difficult problems, we introduce three novel comparison functions which we call *coverage*, *overlap*, and *bundle adjacency*. The first two metrics aim to quantify how well a data reduction performs, and the third quantifies how similar two reductions are to each other. Ideally most points of the full dataset should be near to the reduced set (coverage), but not too many (overlap).

Let *S* and *T* be two sets of streamlines, and let þ > 0 be a selected adjacency threshold. We will say that *s* ∈ *S* is *adjacent* to *T* if there is at least one streamline *t* ∈ *T* with MDF(*s*, *t*) ≤ þ. We define coverage of *S* by *T* as the fraction of *S* that is adjacent to *T*. Coverage ranges between 0 (when all streamlines in *S* are too far from *T*) and 1 (when every streamline in *S* is adjacent *T*).

We define the *overlap* of *T* in *S* as the average number of adjacent streamlines in *T* across adjacent streamlines in *S*. Overlap is undefined if no streamlines in *S* are approximated by *T*, otherwise it can take any value greater then or equal to 1, with higher values indicating the degree of redundancy of *T* in *S*; if *T* has several similar streamlines then this will tend to boost overlap.

Coverage and overlap measure how well one set approximates another. In order to compare two reductions of possibly different data sets we define the symmetric measure *bundle adjacency* (BA). BA is the average of the coverage of *T* by *S* and the coverage of *S* by *T*:

BA(S,T)=(coverage(S,T)+coverage(T,S))∕2.

BA ranges between 0, when no streamlines of S or T have neighbors in the other set, and 1 when they all do.

### 2.5 Data sets

We applied QuickBundles to a variety of data sets: simulations, 10 human tractographies collected and processed by ourselves, and one tractography with segmented bundles which was available online.

#### 2.5.1 Simulated trajectories

We generated 3 different bundles of streamlines from parametric paths sampled at 200 points. The streamlines were made from different combinations of sinusoidal and helicoidal functions. Each bundle contained 150 streamlines. For the red bundle in Figure 4 a pencil of helical streamlines all starting at the same point on a cylinder was generated by linearly varying the pitch of the helices; the green bundle was made up from a divergent pencil of rays on a sinusoidally corrugated sheet; the blue bundle is similarly made from a divergent rays on a sinsusoidally corrugated sheet, with the rays undergoing sinusoidal modulated lateral bending over a range of amplitudes.

#### 2.5.2 Human subjects

We collected data from 10 healthy subjects at the Medical Research Council’s Cognition and Brain Sciences Unit with a 3 T scanner (TIM Trio, Siemens), using a Siemens advanced diffusion work-in-progress sequence, and STEAM (Merboldt et al., 1992; Bernstein et al., 2004) as the diffusion preparation method. The field of view was 240 × 240 mm^2^, matrix size 96 × 96, and slice thickness 2.5 mm (no gap). Fifty five slices were acquired to achieve full brain coverage, and the voxel resolution was 2.5 × 2.5 × 2.5 mm^3^. A 102-point half grid acquisition (Yeh et al., 2010) with a maximum *b*-value of 4000 s/mm^2^ was used. The total acquisition time was 14′21″ with TR = 8200 ms and TE = 69 ms. The experiment was approved by the Cambridge Psychology Research Ethics Committee (CPREC).

For the reconstruction of the 10 human data sets we used Generalized Q-Sampling (Yeh et al., 2010) with diffusion sampling length 1.2 and for the tractography propagation we used EuDX (Euler integration with trilinear interpolation; Garyfallidis, 2012), angular threshold 60° total weighting 0.5, propagation step size 0.5, and quantitative anisotropy stopping threshold 0.0239 (see Figure 9).

#### 2.5.3 PBC human subjects

We also used labeled data sets by experts (see Figures 1 and 5), from the freely available tractography database used in the Pittsburgh Brain Competition Fall 2009, ICDM^1^.

## 3 Results

In this section we justify our claims about the speed and linear complexity of QB (3.1). Next we demonstrate the robustness of QB as a method for clustering tractographies (3.2). In Section 3.3 we show a new way to find similarities across different tractographies and in Section 3.4 we discuss about some potential limitations of our methods and possible workarounds.

### 3.1 Complexity and timings

The execution time of QB is affected by the following parameters: *K*, the fixed number of discretized points per streamline; þ the clustering threshold, which controls the heterogeneity of clusters; and *N* the size of the subset of the tractography on which the clustering will be performed. When þ is higher, fewer more heterogeneous clusters are assembled, and conversely when þ is low, more clusters of greater homogeneity are created.

**Figure 6:**
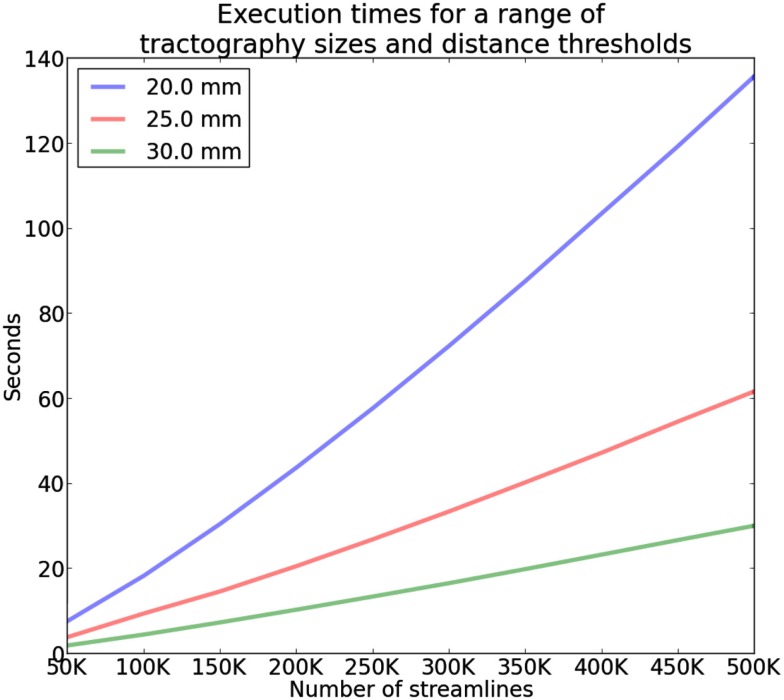
**Time comparisons of QB using different clustering thresholds and different number of streamlines**. Time increases linearly as the number of streamlines increases.

The complexity of QB is in the best case linear time O(N) with the number of streamlines *N* and worst case $O\text{(}N^{2}\text{)}$ when every cluster contains only one streamline. The average case is O(MN) where *M* is the number of clusters however because *M* is usually much smaller than *N* (*M* ≪ *N*) we can neglect *M* and denote it only as O(N) as it is common in complexity theory. We created the following experiment to investigate this claim and we found empirically that the average case is actually O(N) for tractographies (see Figure 6). In this experiment we timed the duration of QB clustering of tractographies containing from 50,000 to 500,000 streamlines in steps of 50,000, with 12 points per streamline and different QB thresholds (20, 25, and 30 mm). These results were obtained using a single thread Intel(R) CPU E5420 at 2.50 GHz on a standard notebook. The results for a single subject can be seen in Figure 6. The same pattern was observed for the remaining 9 subjects. This experiment concludes that QB is suitable for fast and linear time clustering of tractographies.

As a further test we compared QB (with 12 point streamlines and a clustering threshold of 10 mm) with timings reported from the fastest state of the art methods found in the literature. These methods have different goals from those of QB however we think that it is useful to show the important speedup that QB offers for the same number of streamlines. With 1000 streamlines Wang et al.’s (2011) algorithm took 30 s whereas QB took 0.07 s. 14,400 s were required for the same method to cluster 60,000 streamlines; QB took 14.7 s. In a third study a substantial tractography of size 400,000 was clustered by Visser et al. (2011) in 75,000 s; QB completed this task in only 160.1 s. The speedup factors in these three comparisons were 429, 980, and 468, respectively. Therefore, we see the valuable speedup that QB achieves, holding out the prospect of real-time (less than 1 s) clustering on data sets of up to 20,000 streamlines.

### 3.2 Robustness and effectiveness of QB clustering

One of the disadvantages of most clustering algorithms is that they give different results with different initial conditions; for example this is recognized with k-means, expectation maximization (Dempster et al., 1977) and *k*-centers (Gonzalez, 1985) where it is common practice to try a number of different random initial configurations. The same holds for QB so if there are not distinct clusters such that the distance between any pair of clusters is supra-threshold and the diameter of all clusters is sub-threshold, then with different permutations of the same tractography we will typically see similar number of clusters but different underlying clusters. We will examine the robustness of QB in this respect in a number of ways.

[A] First we look at the stability of the number of clusters with respect to random permutations. [B] Next we will use optimized matching agreement (OMA) to establish how well the detailed content of QB clustering is preserved under random permutations. [C] Next we will show using the coverage and overlap metrics how the QB centroids are a better reduction of the tractography dataset than an equivalent number of random selection of streamlines. [D] Finally we will show how well QB clustering on a subset of a tractography dataset serves as an approximation to the remainder of the dataset.

[A] We recorded the numbers of QB clusters in 25 different random permutations of the tractographies of 10 human subjects acquired as described in Section 2.5. Streamlines shorter than 40 mm were first removed and then the remaining streamlines were discretized to 12 points. The mean number of retained streamlines was 98,159.5 (±14,298.0). Then we applied QB with a threshold of 10 mm. The mean number of clusters across subjects was 4,144.0 (±830.0), representing a data compression ratio of about 24:1. There is therefore a considerable between-subject variation in this metric. Some of this inter-subject variability is related to the sizes of the tractographies: the correlation between number of QB clusters and and the size of the full tractography was calculated (*r* = 0.867, *N* = 10, clustering threshold = 10 mm); however the correlation with full tractography size only decreased marginally to *r* = 0.786 when the clustering was performed on subsets of fixed size (80,000). If the number of clusters was more driven by the size of the dataset on which it was based we would expect this correlation to have dropped more. This result suggests that QB clusterings of the subsets reflects the range of shapes of streamlines in the full tractography not solely its size.

By contrast the within-subject variability of the number of clusters across random permutations is rather small, with mean SD 12.7 (min. 7.3; max. 17.4). The standard error of the individual subject means above is (worst case) ±3.9 which gives strong assurance that 25 random permutations are adequate to get reliable subject level estimates and that there is minimal fluctuation across these permutations. This suggests a good level of consistency in the data reduction achieved by QB within each tractography.

[B] Next we investigated how consistent QB clusterings are when datasets are permuted. Sixteen different random permutations were generated for each of 10 tractographies and the corresponding QB clusterings were computed with clustering threshold 10 mm. For each subject the 120 pairings of QB clusterings were compared using the optimized matched agreements index and then averaged. Across subjects the average OMA (see Section 2.4) over the 1,200 comparisons was 72.0% (±0.60%); the average intra-subject SD was ±0.63%. According to Altman (1995) this represents a good level of agreement consistent across tractographies, well above chance.

To motivate our understanding of worst and best case scenarios when the clusterings in question faithfully capture the structure of the underlying dataset, we consider what happens when the dataset consists of parallel lines of uniform spacing. The result of QB clustering is an approximate partitioning into equally spaced pieces. There will typically be an offset between two such partitionings, and the OMA between them will range between 100% when they coincide and 50% when they are most out of phase.

[C] Recognizing that large tractography datasets present a computational challenge, some authors (e.g., O’Donnell and Westin, 2007; Visser et al., 2011) have taken random samples of streamlines (say 10,000) and applied their clustering algorithms to those. We have shown in [A] above that large random subsets do contain much of the information about the full dataset. However they are suboptimal reductions of the full dataset: inevitably they overrepresent the denser parts of the tractography space, and they underrepresent the sparser regions of that space. By their construction the QB centroids will be more uniformly distributed in this space and thus a better simplification of the original dataset. We quantify this using the coverage and overlap statistics (see Section 2.4). Each of the 10 human subject tractographies was split into two halves, the second of which was set aside. QB clusterings were derived for the first halves. The coverage and overlap statistics for the resulting QB centroids and for an equal sized random set of streamlines are presented in Table 1. This was done for two choices of values for the clustering threshold and adjacency threshold: both 10 mm, and then both 20 mm.

**Table 1:** **QB centroids performance compared with random subsets**.

Thresholds	Comparison	Coverage% (SD)	Overlap (SD)
10 mm/10 mm	QB Centroids	99.96 (0.007)	2.44 (0.08)
	Random	90.49 (0.41)	6.16 (0.55)
20 mm/20 mm	QB Centroids	99.99 (0.004)	3.54 (0.18)
	Random	95.86 (0.62)	6.81 (0.93)

We conclude from this that the QB centroids have near perfect coverage, and the typical streamline is adjacent to 2–4 centroids, depending on threshold. By comparison the random subsets have rather lower coverage, failing to approximate between 5 and 10% of the tractography depending on choice of threshold. Moreover the overlap rises strikingly to between 6 and 7. Therefore QB has overall superior performance to a random set.

[D] The final check on the effectiveness of QB clustering centroids is to see how well they approximate a dataset from which they were not derived. For this purpose the coverage and overlap statistics for the QB centroids were compared between the first half of the tractographies from which they were derived, and the second half. The results are presented in Table 2.

**Table 2:** **Performance of QB centroids tested on split halves of datasets**.

Thresholds	Comparison	Coverage% (SD)	Overlap (SD)
10 mm/10 mm	First half	99.96 (0.007)	2.44 (0.08)
	Second half	99.31 (0.08)	2.44 (0.08)
20 mm/20 mm	First half	99.99 (0.004)	3.54 (0.18)
	Second half	99.91 (0.007)	3.54 (0.18)

For each threshold, the first row repeats that of the previous table, while the second row shows that there is only a small fall-off in coverage, and that the overlap is unchanged. The QB centroids are therefore can be taken as a valid reduction of the other halves of the datasets.

### 3.3 Group comparisons

We warped 10 tractographies each belonging to a different healthy subject (see Section 2.5) in MNI space and applied QuickBundles on each tractography independently using clustering threshold 10 mm and discretizing to 18 points. In order to warp the streamlines we first warped FAs from native space to MNI space using the FSL tools^2^. Then we applied the (continuously resampled) displacements to the points in the tractographies in native space in order to warp them to MNI space. The code for doing this is available in http://dipy.org, module dipy.external.fsl, functions create_displacements, and warp_displacements_tracks.

For every subject we only considered the biggest 100 QB clusters, i.e., the clusters which contained the highest number of streamlines. The purpose of this experiment was to identify a similarity measure between the streamlines of the different subjects.

**Figure 7:**
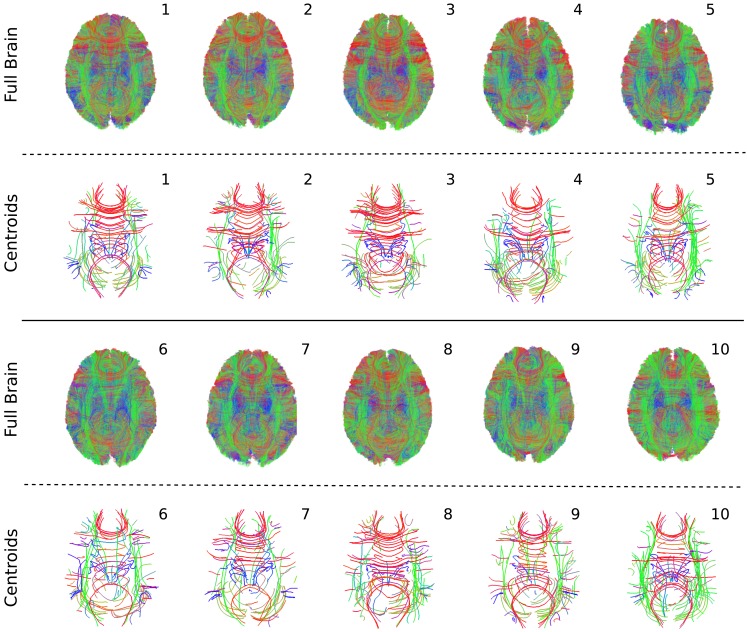
**QuickBundles centroids of the biggest 100 clusters for 10 subjects**. Full tractographies are also presented using high transparency. All streamlines are visualized with the same standard orientation colormap.

In Figure 7 we present both the complete tractographies and the centroid tracks which correspond to the 100 biggest clusters. Because the complete tractographies are very large containing hundreds of thousands of tracks (mean = 171,002.5, SD = 23,879.9) we visualize them using low opacity so that at least an overall projection of the streamlines can be observed. The purpose of this is to show empirically the variability of the streamlines across subjects. By contrast the centroids of the 100 biggest clusters for the 10 subjects are easily observed with full opacity in Figure 7. Each tractography has been substantially simplified by QB such that by visual inspection shows considerable similarities, as well as an interesting range of individual differences. No such visual comparisons could begin to be made based on the whole brain images because the data sets are too dense to draw any conclusions.

**Figure 8:**
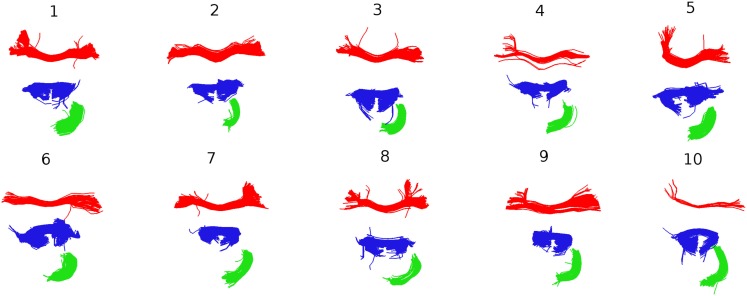
**Following Figure 7, the three largest clusters for subject 1 are shown together with the corresponding clusters in the other nine subjects**. The correspondence is established by finding the centroids in subjects 2–9 that are nearest to the three centroids for subject 1.

Further insights into the kind of correspondences that QB establishes are shown in Figure 8. Taking the clusterings of Figure 7 as the starting point, the centroids belonging to the three largest clusters for subject 1 were identified and the corresponding MDF-nearest centroids were found. For all 10 subjects each of the corresponding clusters is shown. The red and the blue clusters are likely to relate to parts of the corpus callosum while the green clusters indicate parts of the cingulum. Figure 8 gives an impression of the inter-subject variability in the white matter tracts that the centroids represent.

The mean total number of streamlines in the 100 biggest clusters was 4,818.6 (±794.4). These clusters covered on average 16.18% (±1.4%) of the total number of streamlines. We proceeded to use these centroids to study the variability between the streamlines across different subjects.

For this purpose we evaluated the bundle adjacency statistic (BA; see Section 2.4) between all pairs of these 100 centroids of each of the 10 tractographies. This generated 45 BA values with adjacency threshold 10 mm (BA10). We also generated another 45 BA values with adjacency threshold 20 mm (BA20). Note that this latter adjacency threshold for BA is twice that of the initial QB clustering threshold.

For BA10 the most dissimilar subjects were subjects 4 and 6 with BA = 38.5%. The most similar subjects were 4 and 5 with BA = 59.5%. The mean BA10 was 48% (±4.9%). With BA20 the most dissimilar subjects were subjects 7 and 10 with BA = 72%. The most similar subjects were, in agreement with BA10, 4 and 5 with BA = 88.5%. The mean BA20 was 80% (±3.2%).

In this experiment there was a great variability of centroid lengths (mean = 73.6 ± 43.9 mm). If we suppose that shorter streamlines are more likely to be noise artifacts we would expect that by concentrating on longer streamlines we would have a more robust similarity measure for tractography comparison. We propose to follow this up in future work by studying how the length of the big clusters affects BA.

### 3.4 Short streamlines

In general taking short streamlines into account is less valid because (a) the longer streamlines have greater potential to be useful landmarks when comparing or registering different subjects, as they are more likely to be present in most subjects, (b) removing short streamlines facilitates the usage of distance based clustering (no need for manually setting the clustering threshold) and interaction with the tractography, and (c) typically one first wants to see the overall representation of the tractography and later go to the details. MDF distance often separates shorter from longer neighboring streamlines which is both a strength and a limitation according to application. Nonetheless, after having clustered the longer streamlines there are many ways to assign the shorter clusters to their closest longer clusters. For this purpose we recommend using a different distance from MDF for example the minimum version of MAM referred to as MAM_min_ in equation (2).

Here we discuss two simple strategies for clustering short streamlines. The first is an unsupervised technique and the second is supervised.

(1) Cluster the long streamlines using QB with clustering threshold at 10 mm and then cluster the short streamlines (<100 mm) to a lower threshold and assign them to their closest long streamline bundle from the first clustering using the MAM_min_ distance.
(2) Cluster the tractography of a subject, pick a centroid streamline and then find the closest streamlines to that selected streamline using MDF, cluster the closest streamlines found from the previous step and for each one of these new centroid streamlines find the closest streamlines using the MAM_min_ distance. We should now have an amalgamation of shorter and longer streamlines in one cluster.

**Figure 9:**
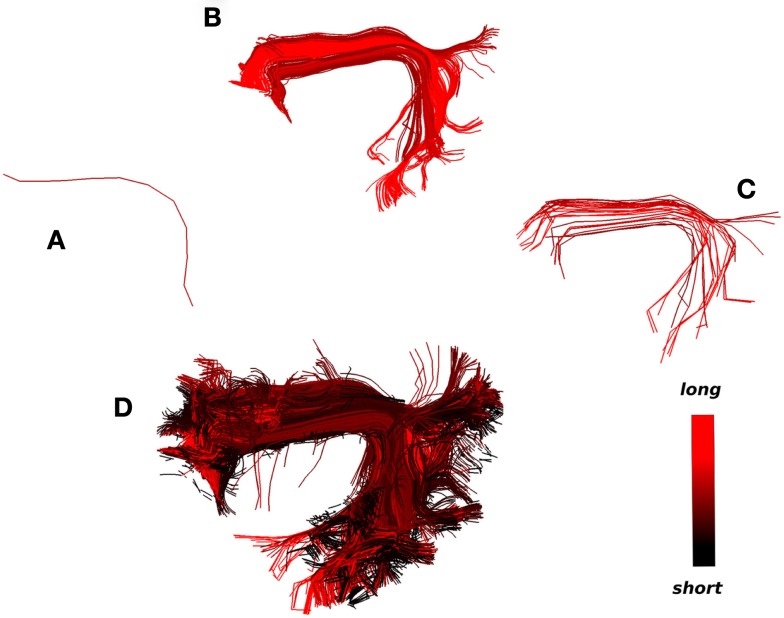
**What could be considered as the strength and limitation of QB is that short streamlines will be clustered differently from longer streamlines although they may belong in the same anatomical bundle**. A solution to this problem is illustrated. The colormap here encodes streamline length. **(A)** A single centroid streamline, **(B)** the 245 actual streamlines closer than 15 mm (MDF distance), **(C)** the streamlines from **(B)** clustered with 23 centroid streamlines using QB with threshold 6.25 mm, **(D)** the 3421 actual streamlines closer than 6 mm (MAM_min_ distance) from the centroid streamlines in **(C)** are shown. We can see that a great number of short streamlines have been brought together along with the streamlines in **(B)**.

An example of this second strategy is shown in Figure 9. A single centroid streamline of interest (Figure 9A) from the region of arcuate fasciculus is selected; the streamlines closer than 15 mm (MDF) to the selected cluster are shown (Figure 9B) and clustered with a distance threshold of 6.25 mm (Figure 9C); finally from every centroid streamline in Figure 9C we find the closest streamlines from the entire tractography (Figure 9D) using the MAM_min_ distance. In this way we managed to bring together in a semi-automatic fashion an entire bundle consisting both of long and short streamlines by just selecting initially a single representative streamline.

## 4 Discussion and Conclusion

We have presented a novel and powerful algorithm – QuickBundles (QB). This algorithm provides simplifications to the problem of revealing the detailed anatomy of the densely packed white matter which has recently attracted much scientific attention; and it is recommended when large data sets are involved. QB can be used with all types of diffusion MRI tractographies which generate streamlines (e.g., probabilistic or deterministic) and it is independent of the reconstruction model. QB is supported by a distance function MDF on the space of streamlines which makes it a metric space. QB can achieve compression ratios of the order of 200:1 depending on the clustering threshold while preserving characteristic information about the tractography.

In common with mainstream clustering algorithms such as *k*-means, *k*-centers, and expectation maximization, QB is not a global clustering method therefore it can give different results under different initial conditions of the dataset when there is no obvious clustering threshold which can separate the clusters into meaningful bundles; for example we should expect different clusters under different permutations of the streamlines in a densely packed tractography. However, we found that there is good agreement even between two clusterings of the same tractography with different permutations. If the clusters are truly separable by distances then there is a global solution independent of permutations. This is often visible in smaller subsets of the initial tractography.

Other algorithms previously too slow to be used on the entire tractography can now be used efficiently too if they start their clustering on the output of QB rather than the initial full tractography.

We saw that QB is a linear time clustering method based on streamline distances, which is on average linear time O(N) where *N* is the number of streamlines and with worst case $O\text{(}N^{2}\text{)}$ when every streamline is a singleton cluster itself. Therefore QB is the fastest known tractography clustering method and even real-time on smaller tractographies (≤ 20,000 streamlines, depending on system CPU). We also showed that it uses a negligible amount of memory.

Additionally, QB can be used to explore multiple tractographies and find correspondences or similarities between different tractographies. This can be facilitated by the use of Bundle Adjacency (BA) a new similarity measure introduced in this paper.

The reduction in dimensionality of the data achieved by QB means that BOIs (bundles of interest) can be selected as an alternative to ROIs for interrogating or labeling the data sets. Our experience with ROI-based matter atlases (WMAs) is that they cannot differentiate fiber directions, i.e., several different bundles could cross an ROI. Therefore, ROIs constructed with a WMA do not lead to anatomical bundles and typically lead to large sprawling sets of streamlines. BOIs seem to be a solution to this problem and BOI creation can be facilitated by QB. Furthermore, we showed that QB can be used to find obscured streamlines not visible to the user at first instance. Therefore, QB opens up the road to create rapid tools for exploring tractographies of any size.

In the future we would like to investigate different ways to merge QB clusters by integrating prior information from neuroanatomists. We are currently working on developing interactive tools which exploit the simplification that QB provides (see Garyfallidis et al., 2012).

We have shown results with data from simulations, single and multiple real subjects. The code for QuickBundles is freely available at http://dipy.org.

## Conflict of Interest Statement

The authors declare that the research was conducted in the absence of any commercial or financial relationships that could be construed as a potential conflict of interest.

## Footnotes

^1^http://pbc.lrdc.pitt.edu

^2^http://fsl.fmrib.ox.ac.uk
